# Author Correction: Demographics and risk of isolation due to sea level rise in the United States

**DOI:** 10.1038/s41467-023-44170-6

**Published:** 2023-12-14

**Authors:** Kelsea Best, Qian He, Allison C. Reilly, Deb A. Niemeier, Mitchell Anderson, Tom Logan

**Affiliations:** 1 Department of Civil, Environmental and Geodetic Engineering, The Ohio State University, Columbus, OH USA; 2 Knowlton School of Architecture, City and Regional Planning, The Ohio State University, Columbus, OH USA; 3 Department of Geography, Planning, and Sustainability, Rowan University, Glassboro, NJ USA; 4 Department of Civil and Environmental Engineering, University of Maryland, College Park, MD USA; 5 Department of Civil and Natural Resources Engineering, University of Canterbury, Christchurch, New Zealand

**Keywords:** Climate-change impacts, Climate-change impacts, Climate sciences

Correction to: *Nature Communications* 10.1038/s41467-023-43835-6, published 30 November 2023

In this article the affiliation ‘Department of Geography, Planning, and Sustainability, Rowan University, Glassboro, NJ, USA’ was incorrectly assigned to Kelsea Best. The original article has been corrected.

