# Supplementary material for: Demographics and risk of isolation due to sea level rise in the United States
Source: Nat Commun. 2023 Nov 30;14:7904. doi: 10.1038/s41467-023-43835-6 (PMC10689761; doi:10.1038/s41467-023-43835-6)
Supplement: Supplementary file 1 — Supplementary Information [file 41467_2023_43835_MOESM1_ESM.pdf]

Demographics and risk of isolation due to sea level rise in the United States

Kelsea Best, Qian He, Allison C. Reilly, Deb A. Niemeier, Mitchell Anderson, Tom Logan

Supplementary Figures

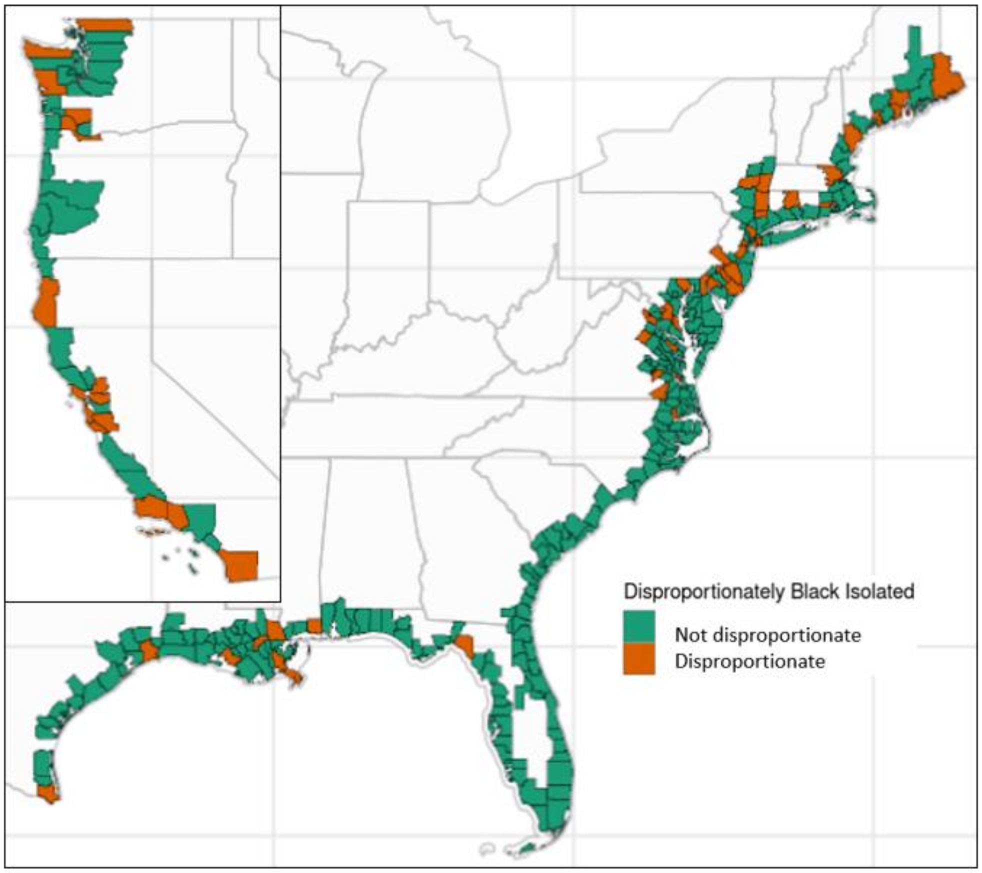

Figure S1: Map of counties where 3 ft of SLR will disproportionately isolate Black populations. Counties where the SLR scenario of 3 ft is predicted to disproportionately result in isolation for Black populations relative to their representation in the county population. Orange indicates disproportionate effects and green represents no disproportionate effect.

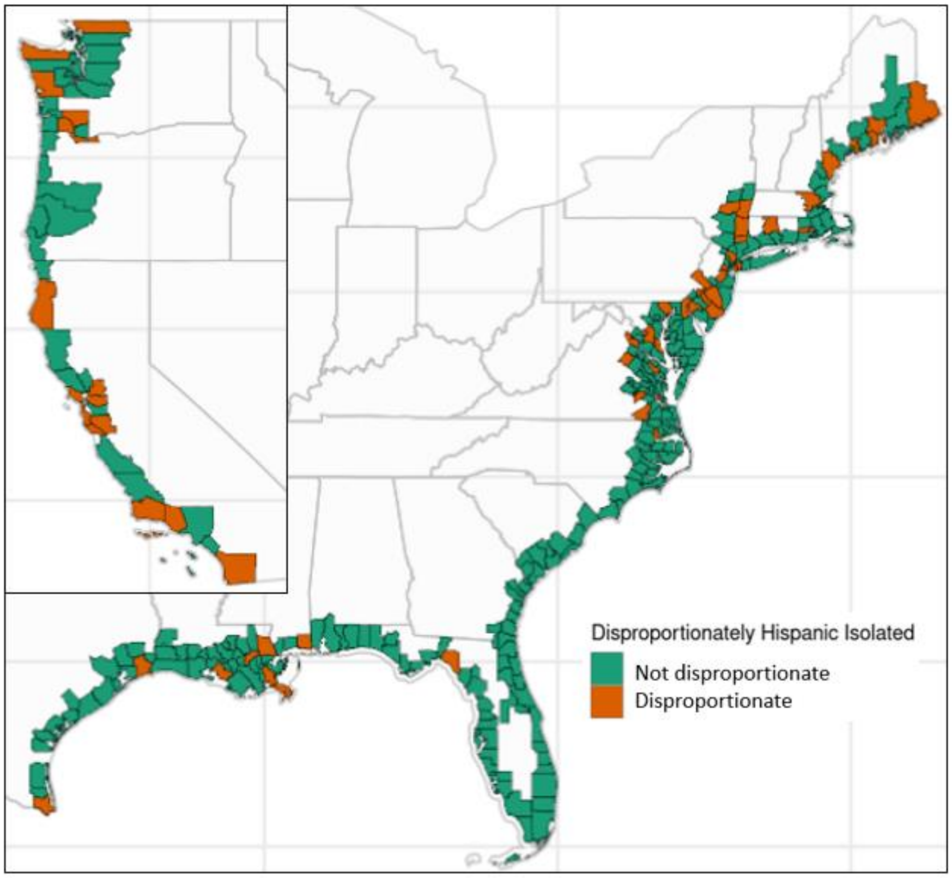

Figure S2: Map of counties where 3 ft of SLR will disproportionately isolate Hispanic populations. Counties where the SLR scenario of 3 ft is predicted to disproportionately result in isolation for Hispanic populations relative to their representation in the county population. Orange indicates disproportionate effects and green represents no disproportionate effect.

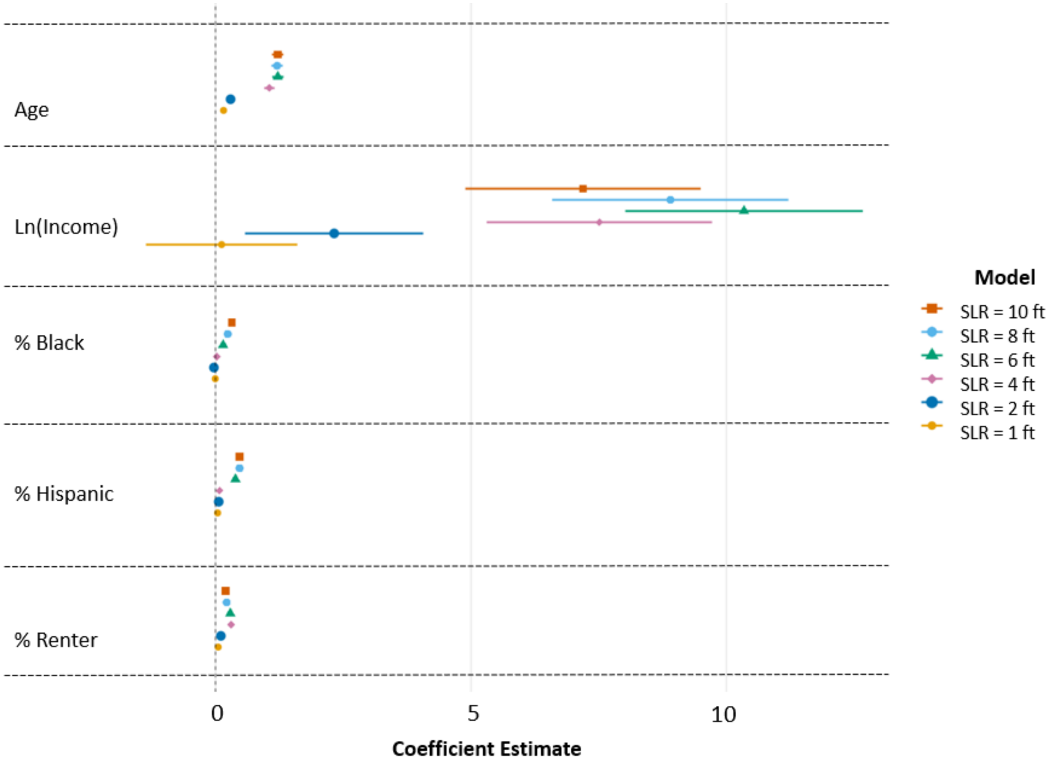

Figure S3: Coefficient plots of individual regressions by level of SLR. Coefficient plots from individual GLM regressions for SLR scenario (1-10 ft). Full results are available in Table S1. Points indicate coefficient estimates, and bars indicate standard errors, presented in Table S1.

Supplemental Methods:

Description of Figure S3: Individual GLM regressions for SLR scenario (1-10 ft).

To understand how the relationship between tract characteristics and isolation risk varies across each SLR scenario, we construct individual Generalized Linear Model (GLM) specifications for a range of SLR scenarios (from 1 to 10 ft) and compare the coefficients across models. Findings from a set of individual Generalized Linear Models (GLM) regressions to predict the fraction of tract population at risk of isolation across 1 to 10 ft of sea level rise suggest that older adults, Hispanic residents, and people of renter status face greater isolation risk in all SLR scenarios. The percentage of the Black population becomes significantly associated with a higher risk of isolation from six feet of SLR. Models at higher SLR scenarios have generally increasing magnitude in coefficients for age, percentage of Black people, and percentage of Hispanic people (**Figure S3, Table S1**).

Supplementary Table:

Table S1: Results of GLMs predicting the percent of population at risk of isolation within a census tract at varying level of SLR (1 to 10 ft). Output from individual GLM models for each SLR scenario from 1 to 10 ft for percent population at risk of isolation in a census tract (%Isolation). All p-values are calculated using a two-sided t-test. Coefficients are also plotted in Figure S3.

	**%Isolation (SLR = 1 ft)**			**%Isolation (SLR = 2 ft)**			**%Isolation (SLR = 4 ft)**			**%Isolation (SLR = 6 ft)**			**%Isolation (SLR = 8 ft)**			**%Isolation (SLR = 10 ft)**		
	**Coef.**	**St. Err.**	**P>z**	**Coef.**	**St. Err.**	**P>z**	**Coef.**	**St. Err.**	**P>z**	**Coef.**	**St. Err.**	**P>z**	**Coef.**	**St. Err.**	**P>z**	**Coef.**	**St. Err.**	**P>z**
Age	0.16	0.031	7.68e-07	0.29	0.039	5.04e-14	1.05	0.051	<2e-16	1.22	0.056	<2e-16	1.20	0.057	<2e-16	1.22	0.057	<2e-16
%Black	-0.0061	0.018	0.74	-0.032	0.021	0.12	0.023	0.026	0.37	0.15	0.027	3.32e-8	0.24	0.026	<2e-16	0.32	0.026	<we-16
%Hispanic	0.041	0.016	0.012	0.062	0.019	0.0011	0.080	0.023	0.00055	0.39	0.023	<2e-16	0.47	0.022	<2e-16	0.47	0.023	<2e-16
Ln(Income)	0.11	0.76	0.87	2.32	0.89	0.0091	7.51	1.12	2.68e-11	10.34	1.19	<2e-16	8.90	1.18	5.07e-14	7.19	1.18	9.74e-10
%Renter	0.051	0.018	0.0042	0.10	0.021	3.73e-07	0.30	0.025	<2e-16	0.29	0.026	<2e-16	0.22	0.026	<2e-16	0.20	0.025	6.31e-15
_cons	-2.72	8.98	0.76	-30.82	10.71	0.0040	-117.34	13.72	<2e-16	-151.54	14.56	<2e-16	-128.56	14.49	<2e-16	-105.08	14.44	3.75e-13
	Obvs = 2087<br>Adj. R^2 = 0.014			Obvs = 3145<br>Adj. R^2 = 0.029			Obvs = 4774<br>Adj. R^2 = 0.096			Obvs = 6044<br>Adj. R^2 = 0.10			Obvs = 6886<br>Adj. R^2 = 0.099			Obvs = 7496<br>Adj. R^2 = 0.094		
