# Supplementary material for: Demographics and risk of isolation due to sea level rise in the United States
Source: Nat Commun. 2023 Nov 30;14:7904. doi: 10.1038/s41467-023-43835-6 (PMC10689761; doi:10.1038/s41467-023-43835-6)
Supplement: Supplementary file 3 — Reporting Summary [file 41467_2023_43835_MOESM3_ESM.pdf]

nature portfolio

Corresponding author(s): Kelsea Best

Last updated by author(s): Nov 11, 2023

Reporting Summary

Nature Portfolio wishes to improve the reproducibility of the work that we publish. This form provides structure for consistency and transparency in reporting. For further information on Nature Portfolio policies, see our Editorial Policies and the Editorial Policy Checklist.

Statistics

For all statistical analyses, confirm that the following items are present in the figure legend, table legend, main text, or Methods section.

n/a	Confirmed	
☐	☒	The exact sample size (*n*) for each experimental group/condition, given as a discrete number and unit of measurement
☐	☒	A statement on whether measurements were taken from distinct samples or whether the same sample was measured repeatedly
☐	☒	The statistical test(s) used AND whether they are one- or two-sided<br>*Only common tests should be described solely by name; describe more complex techniques in the Methods section.*
☐	☒	A description of all covariates tested
☒	☐	A description of any assumptions or corrections, such as tests of normality and adjustment for multiple comparisons
☐	☒	A full description of the statistical parameters including central tendency (e.g. means) or other basic estimates (e.g. regression coefficient) AND variation (e.g. standard deviation) or associated estimates of uncertainty (e.g. confidence intervals)
☐	☒	For null hypothesis testing, the test statistic (e.g. *F*, *t*, *r*) with confidence intervals, effect sizes, degrees of freedom and *P* value noted<br>*Give P values as exact values whenever suitable.*
☒	☐	For Bayesian analysis, information on the choice of priors and Markov chain Monte Carlo settings
☒	☐	For hierarchical and complex designs, identification of the appropriate level for tests and full reporting of outcomes
☒	☐	Estimates of effect sizes (e.g. Cohen's *d*, Pearson's *r*), indicating how they were calculated

Our web collection on statistics for biologists contains articles on many of the points above.

Software and code

Policy information about availability of computer code

Data collection	R/ RStudio v.06.1, tidycensus package version 1.5 in R, geofabrik.de API
Data analysis	Python 3.6, OpenSourceRoutingmachine v5.25.0, RStudio v.06.1, tidyverse package in R, https://github.com/urutau-nz/usa_slr

For manuscripts utilizing custom algorithms or software that are central to the research but not yet described in published literature, software must be made available to editors and reviewers. We strongly encourage code deposition in a community repository (e.g. GitHub). See the Nature Portfolio guidelines for submitting code & software for further information.

Data

Policy information about availability of data

All manuscripts must include a data availability statement. This statement should provide the following information, where applicable:
- Accession codes, unique identifiers, or web links for publicly available datasets
- A description of any restrictions on data availability
- For clinical datasets or third party data, please ensure that the statement adheres to our policy

Data and results for population at risk of isolation are presented on our interactive dashboard https://research.urbanintelligence.co.nz/slr-usa. The remaining data are publicly available and detailed in Methods. These include American Community Survey data (from tidyverse R package v.1.5), OpenStreetMaps (https://www.openstreetmap.org/#map=5/38.007/-95.844), HIFLD (https://hifld-geoplatform.opendata.arcgis.com/), 2020 Census data from IPUMS (https://www.nhgis.org/), NOAA SLR projections, and EvictionLab data v.2.0 (https://evictionlab.org/map/?m=modeled&c=p&b=efr&s=all&r=counties&y=2018&lang=en).

Research involving human participants, their data, or biological material

Policy information about studies with human participants or human data. See also policy information about sex, gender (identity/presentation), and sexual orientation and race, ethnicity and racism.

Reporting on sex and gender	NA.
Reporting on race, ethnicity, or other socially relevant groupings	All human data used was aggregated and publicly available data. We focus this analysis on Black and Hispanic populations, as these populations represent the largest racial minority groups in the U.S. We use "Black" to refer to populations that are Black or African American alone, "Hispanic" to refer to populations that are Hispanic or Latino, and "White" to refer to White alone, not Hispanic or Latino in the American Community Survey data.
Population characteristics	All human data used was aggregated and publicly available. Additional American Community Survey data pulled include median household income, median age, and households living in renter-occupied units.
Recruitment	NA
Ethics oversight	NA

Note that full information on the approval of the study protocol must also be provided in the manuscript.

Field-specific reporting

Please select the one below that is the best fit for your research. If you are not sure, read the appropriate sections before making your selection.

☐ Life sciences ☐ Behavioural & social sciences ☒ Ecological, evolutionary & environmental sciences

For a reference copy of the document with all sections, see nature.com/documents/nr-reporting-summary-flat.pdf

Ecological, evolutionary & environmental sciences study design

All studies must disclose on these points even when the disclosure is negative.

Study description	We evaluate the demographic characteristics (specifically racial composition, age, median household income, percent renter-occupied housing units) of census blocks that are isolated from key destinations under future sea level rise scenarios. The analysis is primarily descriptive.
Research sample	We evaluate all census blocks in the continental U.S. that are in coastal counties (n=299 coastal counties). This sample was selected because all coastal counties are expected to be affected by future sea level rise. Therefore, this sample represents all populations affected directly. Data for these census blocks came from the American Community Survey.
Sampling strategy	The entire population was used.
Data collection	Data was collected from online, public sources using packages in R/RStudio, online APIs, or manually downloading the data from websites (as shapefiles and csv files).
Timing and spatial scale	The data relates to the 2020 US Census and 2019 US American Community Survey. This data is made publicly available by the U.S. Census Bureau. The data used in this work was at the block, block group, tract, and county levels.
Data exclusions	No exclusions.
Reproducibility	This was a statistical summary rather than experimentation.
Randomization	NA- All data within the study area was used.
Blinding	NA- This was not a clinical study. All data was collected from publicly available sources.

Did the study involve field work? ☐ Yes ☒ No

Reporting for specific materials, systems and methods

We require information from authors about some types of materials, experimental systems and methods used in many studies. Here, indicate whether each material, system or method listed is relevant to your study. If you are not sure if a list item applies to your research, read the appropriate section before selecting a response.

Materials & experimental systems

n/a	Involved in the study
☒	☐ Antibodies
☒	☐ Eukaryotic cell lines
☒	☐ Palaeontology and archaeology
☒	☐ Animals and other organisms
☒	☐ Clinical data
☒	☐ Dual use research of concern
☒	☐ Plants

Methods

n/a	Involved in the study
☒	☐ ChIP-seq
☒	☐ Flow cytometry
☒	☐ MRI-based neuroimaging

Plants

Seed stocks	*Report on the source of all seed stocks or other plant material used. If applicable, state the seed stock centre and catalogue number. If plant specimens were collected from the field, describe the collection location, date and sampling procedures.*
Novel plant genotypes	*Describe the methods by which all novel plant genotypes were produced. This includes those generated by transgenic approaches, gene editing, chemical/radiation-based mutagenesis and hybridization. For transgenic lines, describe the transformation method, the number of independent lines analyzed and the generation upon which experiments were performed. For gene-edited lines, describe the editor used, the endogenous sequence targeted for editing, the targeting guide RNA sequence (if applicable) and how the editor was applied.*
Authentication	*Describe any authentication procedures for each seed stock used or novel genotype generated. Describe any experiments used to assess the effect of a mutation and, where applicable, how potential secondary effects (e.g. second site T-DNA insertions, mosiacism, off-target gene editing) were examined.*
